# Multiplicity: an organizing principle for cancers and somatic mutations

**DOI:** 10.1186/1755-8794-4-52

**Published:** 2011-06-29

**Authors:** Lewis J Frey, Stephen R Piccolo, Mary E Edgerton

**Affiliations:** 1 University of Utah, Department of Biomedical Informatics, 26 South 2000 East, Salt Lake City, UT 84112, USA; 2 Huntsman Cancer Institute, 2000 Circle of Hope, Salt Lake City, UT 84112, USA; 3 M.D. Anderson Cancer Center, Department of Pathology, 1515 Holcombe Blvd, Houston, TX 77030, USA

## Abstract

**Background:**

With the advent of whole-genome analysis for profiling tumor tissue, a pressing need has emerged for principled methods of organizing the large amounts of resulting genomic information. We propose the concept of multiplicity measures on cancer and gene networks to organize the information in a clinically meaningful manner. Multiplicity applied in this context extends Fearon and Vogelstein's multi-hit genetic model of colorectal carcinoma across multiple cancers.

**Methods:**

Using the Catalogue of Somatic Mutations in Cancer (COSMIC), we construct networks of interacting cancers and genes. Multiplicity is calculated by evaluating the number of cancers and genes linked by the measurement of a somatic mutation. The Kamada-Kawai algorithm is used to find a two-dimensional minimum energy solution with multiplicity as an input similarity measure. Cancers and genes are positioned in two dimensions according to this similarity. A third dimension is added to the network by assigning a maximal multiplicity to each cancer or gene. Hierarchical clustering within this three-dimensional network is used to identify similar clusters in somatic mutation patterns across cancer types.

**Results:**

The clustering of genes in a three-dimensional network reveals a similarity in acquired mutations across different cancer types. Surprisingly, the clusters separate known causal mutations. The multiplicity clustering technique identifies a set of causal genes with an area under the ROC curve of 0.84 versus 0.57 when clustering on gene mutation rate alone. The cluster multiplicity value and number of causal genes are positively correlated via Spearman's Rank Order correlation (*r_s_*(8) = 0.894, Spearman's *t *= 17.48, *p *< 0.05). A clustering analysis of cancer types segregates different types of cancer. All blood tumors cluster together, and the cluster multiplicity values differ significantly (Kruskal-Wallis, *H *= 16.98, *df *= 2, *p *< 0.05).

**Conclusion:**

We demonstrate the principle of multiplicity for organizing somatic mutations and cancers in clinically relevant clusters. These clusters of cancers and mutations provide representations that identify segregations of cancer and genes driving cancer progression.

## Background

Under the inundation of data from whole-genome analysis of tumors [1], researchers wonder if the aftermath will be a hopelessly complex cancer landscape [2]. For instance, a 2007 study examined somatic mutations across the genome for colorectal carcinoma, one of the most common cancers occurring in epithelial tissue, and observed that a median of 76 non-silent mutations (15 considered to be in candidate cancer genes) had occurred in each tumor [2]. The same study identified a mean of 101 non-silent mutations in a set of breast carcinomas, another common epithelial cancer. As the use of whole-genome sequence analysis increases, the need to organize information about tumor somatic mutation patterns becomes more urgent.

Extant methods [3] to handle the influx of data focus on aggregating data into pathways rather than single-gene focused approaches. The logic of a pathway approach is tied to the observation that gene mutations tend to be exclusive within a pathway [4,5]. The difficulty is that pathways are complex and not mutually exclusive, so defining which pathway a mutation impacts can be open to interpretation; for example, *KRAS *is a member of 34 pathways and *PIK3CA *belongs in 37 [6].

When using genes as the basic element instead of pathways, a method is needed to group genes into mutually exclusive sets that are biologically or clinically relevant. This method reduces the complexity of non-exclusivity that occurs with pathway analysis. The purpose of exclusive sets of genes with somatic mutations is to separate the "wheat from the chaff" or identify which genes are gatekeepers, drivers, or passengers [7,8].

Fearon and Vogelstein introduced a multi-hit genetic model of tumor progression for colorectal cancer pathogenesis based on an accumulation of multiple somatic mutations [9]. This landmark model illustrated that as mutations occur in key genes, normal cells are transformed into communities that proliferate in excess, without adequate apoptosis, and eventually become malignant. The process can be mapped onto histologically recognizable states as colonic tissue progresses from normal mucosa to adenoma (polyp), adenoma with high-grade dysplasia (a more aggressive polyp closer to an invasive state), and finally invasion. Fearon and Vogelstein observed that these mutations typically occur in a recurring sequence, yet the order of the mutations is less important than their cumulative effect.

Our approach extends the concept of multiple hits across cancer types to demonstrate how this type of knowledge representation can be generalized beyond colorectal cancer. Building on Fearon and Vogelstein's multi-hit genetic model of colorectal tumor progression [9], this paper examines the concept of *multiplicity *of genes with somatic mutations across cancers as an organizing principle for analysis of previous and future somatic mutation data. Multiplicity is measured by counting the number of cancers shared by pairs of genes. This gives a measure of similarity between genes based on the number of overlapping cancers between genes. These similarity distances between genes can be used to generate mutually exclusive clusters of genes. The utility of the clusters can be assessed using prior knowledge of cancers and genes.

Multiplicity has been used in social network analysis to identify key actors driving events in capital markets [10]. The social network analysis tool Pajek [10] displays actors' relationships in a three-dimensional landscape, allowing the detection of patterns not visible on a two-dimensional surface. By treating genes and cancers as actors and events using social network analysis techniques, we found evidence of patterns in cancer and gene multiplicity landscapes.

The following sections discuss two experiments that examine the concept of multiplicity. The first study tests whether high multiplicity scores correlate with known causal genes; it also explores the use of multiplicity combined with hierarchical clustering to organize mutated genes. The second study examines the utility of multiplicity in clustering cancer types.

## Methods

Experiment 1 examines the utility of multiplicity and clustering methods for mutated genes. A clustering hierarchy is generated with mutated genes as the focus. We then compared the generated clusters to the Wellcome Trust Sanger Institute's list of known germline mutations [11] to test for associations related to multiplicity and known causal mutations.

### Data

Over past decades, hundreds of thousands of tumor samples have been examined for somatic mutations and reported in the literature. Consequently, thousands of somatic mutations have been linked to hundreds of different cancer types [12]. Researchers at the Wellcome Trust Sanger Institute have manually curated somatic mutation information available through research publications into a public database called the Catalogue of Somatic Mutations in Cancer (COSMIC) [13,14]. We use hierarchical clustering of somatic mutation profiles randomly sampled from the COSMIC resource to identify high multiplicity similarities in somatic mutation patterns across cancer types.

For our analysis, we use the July 2010 COSMIC (v48) containing data for 18,490 genes, 136,326 mutant tissue samples, and 2,720,220 experiments. In those samples, 141,212 unique mutations are identified. These data are extracted from 10,383 articles published between 1984 and 2010. The curators for the COSMIC database point out that the classification of tumor types in the published literature is variable both in terms of the level of granularity provided in a single publication and in terms of the prevailing classification system used for a particular cancer type. To standardize the tissue and histology information stored in their database, COSMIC curators developed a tumor classification scheme using generally understood terminology. They translate tissue and histology information into the COSMIC classification scheme before entering it into the database. A mapping of published terminologies and the COSMIC classification scheme is available from the Sanger website [14].

We first defined *cancer type *as a combination of COSMIC information that informed us of the tumor's anatomic site or tissue type of origin and the tumor's histology. We selected the most granular histological classifier for which we were able to maintain sufficient sample size to provide power for the analysis while adequately classifying the disease for biological interpretation.

We selected the "Site_Primary_COSMIC" value for all anatomic site or tissue types represented. In general, COSMIC contained data on too few samples with designated subtypes for tumors of epithelial origin to classify them beyond histology of adenoma or carcinoma. Therefore, for tumors of epithelial origin we concatenated the COSMIC anatomic site of a tumor (called "Site_Primary_COSMIC") with the highest-level histological classifier (called "Histology_COSMIC") to define cancer types. We used a similar concatenation for soft tissue tumors, categorizing the tumor types using the highest level histology available (e.g., gastrointestinal stromal tumors).

In contrast, we found that the highest level histological classifier separated tumors of the "hematopoietic and lymphoid" tissue type into two large groups, either "hematopoietic neoplasm" or "lymphoid neoplasm." In these cases, we used the histological subtype (e.g., "chronic myeloid leukemia") to classify the tumor type because it more accurately represents the tumor type, and there are sufficient samples to make the analysis meaningful. Similarly, with central nervous system tumors, we were able to group the gliomas into subtypes (e.g., astrocytoma grade IV).

Gene substitutions, insertions, and deletions are classified uniformly as *mutations*. In some genes (e.g., *JAK2*, *KRAS*, *PDGFRA*), mutations are focused mainly within a few key sequence regions; in others (e.g., *APC*, *TP53*, *CDKN2A*), infrequent mutations are spread across hundreds of positions within the gene. To maintain computational feasibility and in an attempt to represent higher-level biological effects, we aggregate mutations at the gene level rather than considering each mutation as a distinct feature.

In preliminary work, we found that differences in the sample sizes for cancer types resulted in the larger samples having more weight in the clustering. This, along with the selection bias for the measured genes, makes normalization through clustering difficult. For this investigation, we forced equal sample sizes (N = 4000) by restricting our investigation to cancer types with 4,000 or more samples in COSMIC, randomly sampling 4,000 mutant tissue samples per cancer type from COSMIC. The analysis uses the closed-world assumption of databases [15], where a mutation exists if and only if there is positive evidence for the mutation.

#### Known causal genes

COSMIC also includes a listing of known causal germline gene mutations. We obtained the counts for the known causal gene mutations by intersecting Sanger Institute's 75 known germline mutation genes [11] with the 489 genes included in the network. This results in an intersecting set of 34 genes. We acknowledge many other causal genes identified in the literature, but our analysis focuses on Sanger Institute's recognized causal germline mutations. Additionally, these germline mutations have been identified through genetic linkage studies of cancer pedigrees [16] and are used as a means of early detection and treatment of cancers through screening.

### Hierarchical clustering technique

We used the Ward hierarchical clustering technique [17] as implemented in Pajek [10] to cluster data using the Euclidean distance between genes. The generation of the clusters involves several steps: construct two-mode cancer and gene network, produce one-mode gene network and one-mode cancer network, calculate gene multiplicity values, generate Kamada-Kawai two-dimensional plane, compute 3D similarity matrix, and find hierarchical clusters.

#### Two-mode network for cancers and genes

**Figure 1:**
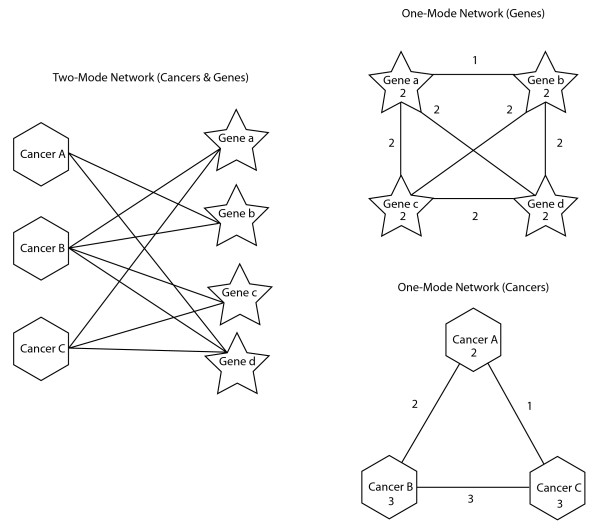
**Examples of two-mode and one-mode networks**. The example two-mode network on the left side has links only between cancers and genes. On the right side, one-mode networks of genes (upper) or cancers (lower) are derived from the two-mode network. The number on each link corresponds to the number of shared cancers between the genes in the upper network and the number of shared genes between the cancers in the lower. The number within each node is the maximal value for the links connected to the node.

A network is constructed with nodes being cancers types and genes, and links being the relationship between cancers types and genes. This results in a two-mode network with cancers being one of the modes and genes being the other. Cancers within the two-mode network are associated only with genes and genes are associated only with cancers (Figure 1, left side). For the two-mode network generated from COSMIC data, a link between a mutated gene and a cancer type is included in the network if the gene's mutation rate for a given cancer type is better than chance as tested by a chi-square goodness-of-fit test (*p *< 0.05). This results in 29 types of cancer and 489 genes with mutations in the network.

#### One-mode network for genes

The two-mode graph is converted to a one-mode graph (Figure 1, top right side) of multiplicity-related genes in which the multiplicity of the link represents the number of cancers in which the linked genes have occurred together. As illustrated in Figure 1, genes *a *and *b *share links only through Cancer *B*, so their *link *multiplicity is one.

#### Gene maximum multiplicity value

A multiplicity-slice (*m*-slice) [10] is then applied to the graph, giving a multiplicity value to each gene in the network derived from the maximal multiplicity of the links within a gene's set of directly connected genes. For example in Figure 1, a gene maximum multiplicity value of 2 is listed inside all the nodes since each node has a maximum multiplicity link of magnitude 2.

Specifically, gene maximum multiplicity (Eq. 1, GeneMaxMultiplicity) and gene link multiplicity (Eq. 2, GeneLinkMultiplicity) are defined as follows. Let *G *be the set of genes, *C *be the set of cancers, and *C'_ij _*be the set of cancers such that both genes *i *and *j *are mutated in cancer *k *more than chance as measured by chi-squared at alpha 0.05.(1)(2)

Thus, gene maximum multiplicity is larger for genes that occur systemically across cancers. Gene maximum multiplicity serves as a measure of a gene's systemic occurrence and hence a gene's potential to be a driver mutation rather than a passenger mutation.

#### Kamada-Kawai two-dimensional plane

The gene network is then arranged in a two-dimensional space using the Kamada-Kawai network drawing algorithm [18] implemented in Pajek [10]. The algorithm arranges the genes according to minimum energy of the system with the links between genes being analogous to springs [18]. In Figure 1, Genes *c *and *d *are more similar to each other, with a gene link multiplicity value of *2*, than Genes *a *and *b*, with a gene link multiplicity of 1. Consequently, Genes *c *and *d *would be closer to each other in the two-dimensional space solved for minimum energy.

The maximum multiplicity value of each gene is used as a third-dimension perpendicular to the Kamada-Kawai two-dimensional plane. Thus, each gene has an associated *x*, *y *and *z *coordinate based on its position in the Kamada-Kawai plane and its gene maximum multiplicity value.

#### Three-dimensional similarity matrix and hierarchical clustering

We computed a similarity matrix by calculating the Euclidean distance between the three-dimensional coordinates for each gene in the network. We then used the similarity matrix to construct the hierarchical clustering given the distances between the genes. This Euclidean distance clustering approach overcomes the cluster instability limitation due to sparse data reported with previous methods applied to COSMIC [19].

### Network method for Experiment 2

Experiment 2 applies the same methods as Experiment 1, but focuses on cancer types rather than mutated genes. The approach constructs a network of cancer types with a cancer maximum multiplicity measure establishing the similarity and dissimilarity between cancer types. Given cancer maximum multiplicity derived distances, a hierarchical clustering technique is used to generate meaningful clusters. Experiment 2 uses the same data sets as Experiment 1. The data sets and selection process are described in Experiment 1.

Experiment 2 uses the same graph methodology as Experiment 1, performed for cancer types rather than mutated genes. The one-mode graph links cancers in which the multiplicity of the links between the cancer types represents the number of genes they share. Similar to Experiment 1 for gene maximum multiplicity, the cancer maximum multiplicity value for a cancer type is obtained from its maximum value link to any cancer type in the graph. Specifically, cancer maximum multiplicity (Eq. 3, CancerMaxMultiplicity) and cancer link multiplicity (Eq. 4, CancerLinkMultiplicity) are defined as follows:(3)(4)

## Results

For Experiment 1, we have generated five results for mutated genes using the above network representation: (1) a two-sample Kolmogorov-Smirnov test to show whether higher gene maximum multiplicity values occur with known causal genes, (2) a paired histogram comparing average gene maximum multiplicity of clusters with counts of known causal genes within clusters, (3) a dendrogram of gene clusters generated by hierarchical clustering, (4) a clustering of germline mutations only, and (5) a comparison of the different measures for clustering the somatic mutation data.

### Gene maximum multiplicity and causal genes

The observed gene maximum multiplicity values for each *gene *ranged from 1 to 23. Overall, genes with high gene maximum multiplicity scores were significantly more likely to be causal genes than those with lower gene maximum multiplicity scores (two-sample Kolmogorov-Smirnov test, one-tail, *D*_34, 455 _= 0.632, *p *< 0.05). The median gene maximum multiplicity score for known causal genes was 5.5; for all other genes, it was 1.0.

### Gene clusters

**Figure 2:**
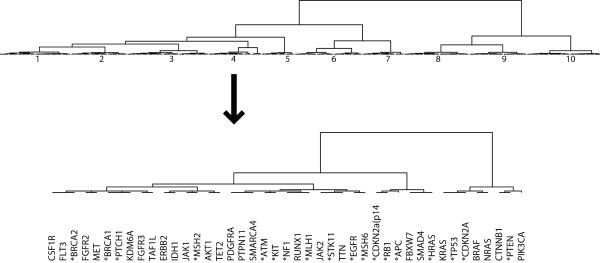
**Graphical depiction of the gene clustering**. The clustering of the 489 genes derived from their Euclidean distances in the Kamada-Kawai and gene maximum multiplicity three-dimensional space. The dissimilarity distance calculated in the Ward hierarchy ranges from 0.0 to 41.09. The dissimilarity threshold for the clusters is 5.07. The lower portion of the figure depicts cluster 4, which has the highest gene maximum multiplicity value of 10.3. The asterisks mark the known causal gene mutations within this cluster.

The Ward hierarchical clustering technique [10,17] resulted in ten clusters (see Experiment 1 description of technique). The dissimilarity threshold of 5.07 was chosen from inspection of the dendrogram for a cluster cut point. The clusters are depicted in Figure 2. Cluster 4 has the highest average multiplicity of 10.3. The lower portion of Figure 2 provides a detailed view of cluster 4. Note that this cluster contains 18 known causal gene mutations (see asterisks in Figure 2, cluster 4).

### Average gene maximum multiplicity for clusters and causal genes

**Figure 3:**
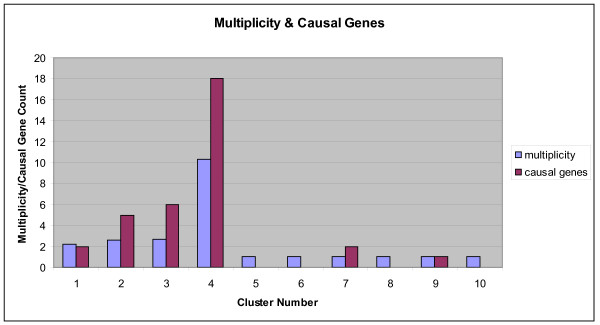
**Histogram for the average gene maximum multiplicity value for the clusters**. The histogram depicts the average gene maximum multiplicity values for the ten clusters derived from the hierarchical clustering method presented in Figure 2. The average gene maximum multiplicity for a cluster is obtained by averaging the gene maximum multiplicity values of the genes within a cluster. The causal genes are tallied for each mutually exclusive cluster to generate the histogram of causal gene counts per cluster.

Figure 3 displays the histogram showing the average gene maximum multiplicity value for the ten clusters derived from the hierarchical clustering method depicted in Figure 2. The average gene maximum multiplicity for a cluster is obtained by averaging the gene maximum multiplicity values of the individual genes within a cluster. The observed gene maximum multiplicity values for each *cluster *range from 1.0 to 10.3 (see Figure 3). We tallied the causal genes for each mutually exclusive cluster to generate the histogram of causal gene counts per cluster. The number of *causal genes *observed in each cluster ranges from 0 to 18 and is presented in Figure 3.

A disproportionate allocation of causal gene mutations occurs within clusters having a high average gene maximum multiplicity value. Clusters 1, 2, 3 and 4 all have average gene maximum multiplicity values greater than 2.0, and they have a disproportionate number of known *causal gene *mutations (31 of 34). Cluster 4, with 18 causal mutations, is the highest-ranking cluster, with an average gene maximum multiplicity of 10.3. Cluster 4 contains more than half the intersecting casual mutations. There is a strong positive correlation between average gene maximum multiplicity values for clusters and number of causal genes per cluster (*r_s_*(8) = 0.894, Spearman's *t *= 17.48, *p *< 0.05).

### Three-dimensional gene representation

**Figure 4:**
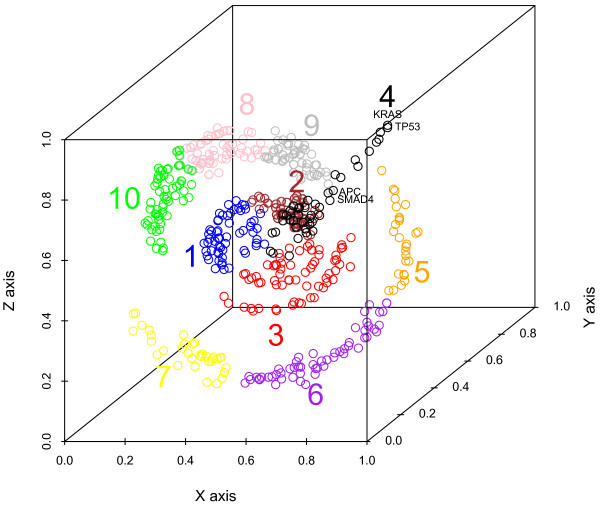
**Three-dimensional gene maximum multiplicity representation**. The three-dimensional representation of cancer is derived from the combination of the *x *and *y *dimensions of the Kamada-Kawai network with a *z*-dimension for the gene maximum multiplicity values. Each color denotes one of the ten clusters. The clusters are derived from the Euclidean distances between nodes in the Kamada-Kawai network layout with a perpendicular gene multiplicity dimension. The color-coded numeric label for each cluster is listed near the points for that cluster.

Figure 4 displays the three-dimensional layout with colors representing each cluster. Cluster 4 (black symbols) projects on a central axis perpendicular to the two-dimensional plane of the Kamada-Kawai layout. This cluster contains the greatest number of known causal genes and has the highest multiplicity values.

The three-dimensional representation reveals additional information beyond the driver genes alone. For example, clusters 8, 9 and 10 are all associated with astrocytoma grade IV, and the genes have clustered together tightly. Cluster 5 primarily has genes associated with ovarian cancer, cluster 6 has a large proportion of genes associated with breast and lung cancers, and cluster 7 has a large proportion of genes associated with breast and central nervous system cancers. Cluster 3 has systemic genes that occur across many of the cancers and a concentration of genes associated with the blood cancers. Clusters 1 and 2 have genes that are primarily associated with carcinomas. Cluster 4 has systemic genes that occur across many of the 29 cancers. Cluster 4 also contains all but 1 of the 24 genes associated with the precancerous tumors: large intestine adenoma, thyroid adenoma, and gastrointestinal stromal tumor. The simpler one-dimensional measure, gene maximum multiplicity alone, provides none of this information.

### Clustering of germline mutations only

Clustering only on the 34 causal germline mutations results in pattern similar to Figure 4 but with fewer genes. Additional File 1 presents a graphical representation of the hierarchical clustering for these 34 causal germline mutations.

### Comparison of causal gene prediction

We compared four different measures for clustering the somatic mutation data: (1) clustering on the three-dimensional combination of the Kamada and Kawai and gene maximum multiplicity, (2) clustering on the one-dimensional representation of gene maximum multiplicity, (3) clustering on the two-dimensional Kamada and Kawai representation, and (4) clustering on the one-dimensional representation of the genes' mutation rates. We calculated thresholds for predicting germline genes by the cluster's average gene maximum multiplicity value divided by the maximum of these average values over the clusters. The same method is applied for mutation rate, with average mutation rate for a cluster being substituted for average gene maximum multiplicity rate. The results for the four comparisons are 10, 3, 9, and 5 clusters, respectively. The number of clusters for each comparison was determined by the distance between clusters in each dendrogram. As shown in Table 1, three-dimensional clustering performs the best overall, with 0.84 area under the receiver operating characteristic (ROC) curve. This is only a 0.01 improvement over the one-dimensional gene maximum multiplicity clustering, so gene maximum multiplicity is the primary measure generating the clusters that differentiate germline mutations from non-germline mutations. Contrast this with mutation rate, 0.57 area under the ROC, accounting for 21 of the 34 germline mutations in the top 489 genes. Mutation rate results in a maximum sensitivity of only 62% of the genes for the germline comparison. The clustering on two dimensions performs poorly compared to the three-dimensional and one-dimensional clusterings. It creates a large cluster in the center in the Kamada-Kawai, which does not distinguish well between germline mutations and non-germline mutations.

**Table 1:** Comparison of area under ROC for measures

Measures	Dimension	Area under ROC
Kamada-Kawai and gene maximum multiplicity	3D	0.84

Gene maximum multiplicity	1D	0.83

Kamada-Kawai	2D	0.79

Mutation rate	1D	0.57

Comparison of four different measures for clustering the somatic mutation data: (1) a clustering which combines the two-dimensional representation of the Kamada and Kawai and gene maximum multiplicity, (2) a clustering on the one-dimensional representation of gene maximum multiplicity, (3) a cluster on only the two-dimensional Kamada and Kawai representation, and (4) a clustering on the one-dimensional representation of mutation rates of the genes

The result listed in Table 1 supports using the simpler one-dimensional measure of gene maximum multiplicity to predict germline mutations. However, although the gain in area under the ROC is slight, the three-dimensional graph representation provides valuable spatial information. Additional File 2 provides a gene maximum multiplicity value for each gene.

In the COSMIC data set, there is a confounding influence of bias in which commonly measured genes tend to be those known to be associated with cancer progression. Clinicians and researchers use prior knowledge to select genes for measurement that they think are most relevant to the particular cancer. Consequently, one could argue that gene maximum multiplicity merely capitalizes on a bias in the data set. To address this concern, we examined the correlation between the number of measurements on specific genes and the gene maximum multiplicity measure on those genes. A significant positive correlation was found between gene maximum multiplicity and the frequency of gene measurement (*r_p_*(487) = 0.74, Pearson's *t *= 23.9, *p *< 0.05). With an R^2 ^of 54.8%, this suggests that about 55% of the variance might be explained via the frequency of gene measurement. The multiplicity measure not only successfully identifies genes already known to be important; it also gives high ranking to less frequently measured relevant genes (see Table 2).

**Table 2:** Large multiplicity values for genes that have a low frequency of measurement

Gene	Gene maximum multiplicity value	Number of times measured in analyzed data set
CDKN2a(p14)	14	782

TTN	9	462

SMARCA4	7	1307

CSMD3	5	983

TAFIL	5	783

MTOR	4	941

Although there is a high correlation, a number of genes have above-average gene maximum multiplicity values (average = 2.3) despite having a below-average number of measurements (average = 1813.7). This table shows the subset of genes with these characteristics.

This suggests that prior knowledge selection bias misses genes systemically mutating across cancers that the gene maximum multiplicity measure can identify. Although the COSMIC data set has limitations, the gene maximum multiplicity measure can be used to find candidate genes for investigation as systemic somatic mutations. The measurement bias exhibited in COSMIC can be further examined when whole genome sequence data repositories for cancer [20] become sufficiently populated to apply the gene maximum multiplicity measure.

### Biological implications of the gene clusters

The highest gene maximum multiplicity cluster, cluster 4, has a number of known genes associated with the development of multiple types of cancer. For example, it includes *KRAS*, *APC*, *SMAD4 *and *TP53*, genes from the Vogelstein model of colorectal cancer progression [9,16,21]; *RB1 *and *NF1*, well-known tumor suppressor genes [3]; *MET *and *PDGFRA*, which are not listed in the Sanger Institute file for germline mutations, but are known to be causal for hereditary papillary renal cell carcinoma and familial gastrointestinal stromal tumors, respectively [3]; *BRCA1 *and *BRCA2*, both known to be causal in the development of breast and ovarian cancer [22,23]; and *EGFR *and *ERBB2*, both members of the epidermal growth factor receptor family and known to have roles in promoting proliferation and aggressive behavior. In addition to depicting known causal genes, the method implicates by association many other genes that that occur across multiple cancers, suggesting their possible significant role in cancer progression. These implicated genes include *TTN*, *BRAF*, *ATM*, and *TAF1L*, the top four protein kinase genes ranked by Greenman and colleagues [8] as carrying at least one driver mutation. This finding supports the logic of using gene maximum multiplicity as a key feature in our analysis. In addition to organizing the data, this method allows us to hypothesize that the other genes in cluster 4 have a high probability of a causal role in progression.

Clusters 1, 2, and 3 are at cluster 4's base and contain a total of 13 known causal genes. These three clusters have the next highest gene maximum multiplicity values. The remaining clusters form a circular pattern at the periphery and contain a total of 3 known causal genes. Genes with higher gene maximum multiplicity values will cluster more tightly on the central axis. This is evident with *TP53*, *KRAS *and *CDKN2A*, which have the highest gene maximum multiplicity values and appear at the tip of the central axis. The central axis can be thought of as a common set of generally applicable gene mechanisms for driving carcinogenesis in any cell type. The higher the gene maximum multiplicity value, the more broadly the gene occurs across the 29 cancers in the network. At the base, genes that occur in subsets of cancer types begin to fan out from the axis. Here we begin to see genes specific to a certain cancer type or anatomic location. We note that our data are biased toward cancer types that include fully invasive or malignant tumors, especially carcinomas. Therefore, the genes that fan out from the axis tend to be associated with the invasive state.

### Three-dimensional cancer representation

For Experiment 2, we generated the following three representations for cancer types: (1) three-dimensional space of cancer types from which the clusters are derived based on Euclidean distance; (2) a dendrogram of specific cancer types within the clusters generated using hierarchical clustering; and (3) a dendrogram resulting from normalizing the cancer link multiplicity by the mutation rate for the cancers connected by the link.

**Figure 5:**
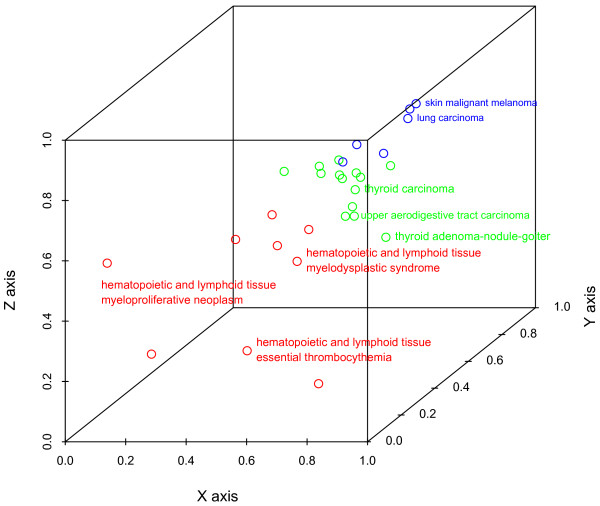
**Three-dimensional cancer maximum multiplicity representation**. The three-dimensional representation of cancer is derived from the combination of the *x *and *y *dimensions of the Kamada-Kawai network with a *z*-dimension for the cancer maximum multiplicity values. The colors represent the three clusters generated from the Euclidean distances between cancer types as described in the text. Cluster 2, depicted in red, contains only hematopoietic and lymphoid tissue tumors. Specific cancers depicted in the green, red and blue clusters are listed in Figure 6 under clusters 1, 2 and 3, respectively.

Figure 5 displays the three-dimensional space derived from the Kamada-Kawai two-dimensional graph layout, with the cancer maximum multiplicity representing the third dimension. The cancer maximum multiplicity values of the cancers range from 4 to 53. The coordinates are normalized to be within a unit space. The minimum energy solution for the network results in cancers with many links to other cancers being centrally located in the two-dimensional space. The hematopoietic and lymphoid tissue tumors share similar mutations and group together in the three-dimensional space (See Figure 5, red symbols and labels).

Most tumors in COSMIC, with over 4000 samples, are fully invasive carcinomas. Thus, our analysis contains a relatively small number of tumors that are either precursors to an invasive state or are not frankly malignant (e.g., adenomas, in situ carcinomas, and tumors with unknown malignant potential). Of the data used, the precancerous or not frankly malignant tumors and adenomas (n = 3) tend to have lower cancer maximum multiplicity values and appear in the same cluster (see Figure 5, green symbols). The average cancer maximum multiplicity values for clusters 1, 2, and 3 are 14.5, 8.11, and 26.5, respectively. The ranks of the average of the cancer maximum multiplicity values for clusters are significantly different via a Kruskal-Wallis test (*H *= 16.98, *df *= 2, *p *< 0.05). Median multiplicity value for clusters 1, 2, and 3 are 15, 10, and 45, respectively.

**Figure 6:**
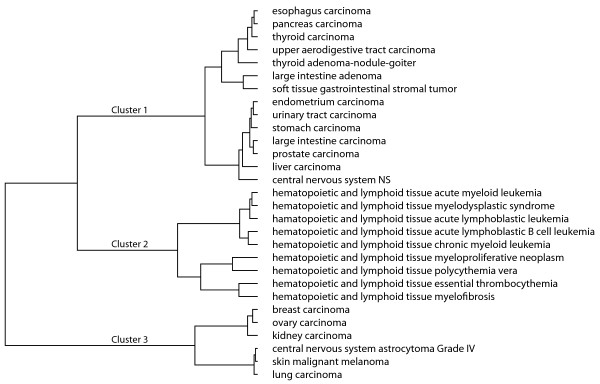
**Graphical depiction of the cancer clustering**. The clustering of the 29 cancer types derived from their Euclidean distances in the Kamada-Kawai and cancer maximum multiplicity three-dimensional space. The dissimilarity distance calculated in the Ward hierarchy ranges from 0.0 to 3.42.

We used the Ward hierarchical clustering technique [10,17] to cluster the data using the Euclidean distance between cancer types. Figure 6 focuses on the three high-level clusters of cancer type. Hematopoietic cancers cluster together (see red symbols in Figure 5). Lung cancer, malignant melanoma, and central nervous system astrocytoma grade IV, all aggressive cancers, cluster tightly. These cancers have the highest cancer maximum multiplicity values (see blue symbols in Figure 5). Breast, ovarian, and kidney cancers are also included in the high cancer maximum multiplicity cluster. The third cluster has a mixture of precancerous adenomas and tumors, along with carcinomas (see green symbols in Figure 5).

By normalizing the link weight between cancers based on the cancer's mutation rate, we obtained a clustering less influenced by the number of mutations occurring within each cancer. For example, astrocytoma grade IV has 299 mutated genes associated with it, while large intestine adenoma has 10 mutations. The mutation rate of a cancer does give information about the aggressiveness of the cancer due to genetic instability. Astrocytoma grade IV is in a much worse state than adenoma of the large intestine. To normalize the link between adenoma of the large intestine and astrocytoma, we set the weight that each one contributes as proportional to the inverse of its overall number of mutations, so adenoma of the large intestine would be weight 1/10 and astrocytoma grade IV would be 1/299.(5)

**Figure 7:**
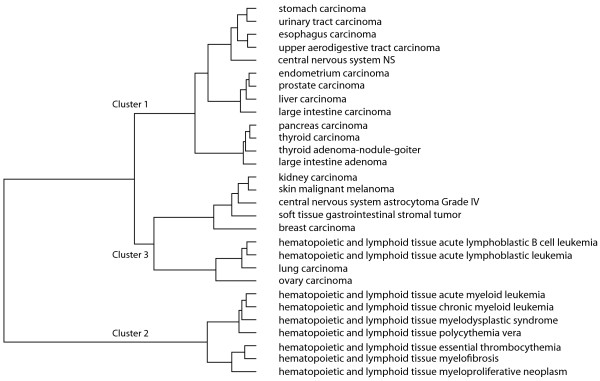
**Graphical depiction of the normalized cancer clustering**. Using normalized link weights, the clustering of the 29 cancer types derived from their Euclidean distances in the Kamada-Kawai and cancer maximum multiplicity three-dimensional space. The dissimilarity distance calculated in the Ward hierarchy ranges from 0 to 2.85.

Equation 5 provides the formulation of the normalized link weight with *|| i || *being the number of mutated genes associated with cancer *i*. Figure 7 shows the hierarchical clusters that result from clustering with normalized link weights.

Comparing Figure 6 to Figure 7, the overall structure of the clustering hierarchy is similar to the non-normalized clusters. Three main clusters with high mutation rate cancers remain together. The blood cancers still cluster together under the normalization, but two acute blood cancers, more aggressive and therefore more unstable with respect to a unique genetic identity, cluster with two other cancers that have high mutation rates, lung cancer and ovarian carcinoma. In the non-normalized clustering, these two acute blood cancers clearly cluster only with related blood cancers because lung and ovarian cancers have high mutation rates and have a larger distance from the blood cancers due to high mutation rates. Normalization may in fact diminish the distance between cancers, blurring of the boundaries between certain cancer types. It may be that clustering cancers by multiplicity and Euclidean distance is more informative without the normalization step.

### Biological implications of cancer type clusters

The biological basis of the strong central cluster of hematopoietic tumors (Cluster 2) is straightforward. Clusters 1 and 3, which each contain several tumor types, require a closer examination of their associations. One subcluster in cluster 3 contains Grade IV astrocytomas, malignant melanomas, and lung carcinomas. Astrocytomas and melanomas both originate from neural crest tissue, as do the subset of lung carcinomas that are either pure neuroendocrine carcinomas (such as small cell carcinoma of the lung) or contain neuroendocrine differentiation. It is also known that *EGFR *mutations or amplifications play a role in lung adenocarcinomas [24,25], uveal melanomas [26], and possibly in high-grade gliomas [27], although the role *EGFR *plays with respect to clinical behavior of the tumor has been controversial [28].

In the first part of this paper, *EGFR *can be located on a central axis of genetic mutations with the highest gene maximum multiplicity (see Figure 2), and that therefore may represent an axis of genetic instability that leads to progression into and across neoplastic states from a proliferating clone to a frankly malignant tumor. These tumor types may cluster based on genetic instability, possibly introduced or associated with *EGFR *amplifications. The relationship with neural differentiation is not clear.

The second subcluster of cluster 3 includes breast, ovarian, and kidney carcinomas. Relationships between breast and ovarian carcinomas have been established, including association with a germline mutation in *BRCA2 *and the influence of reproductive steroid hormone receptors. The association with kidney carcinomas is novel in this analysis, but may be due to the fact that a number of urothelial carcinomas express Erbb2 (also known as Her2neu [29]), which interacts with *EGFR *and may introduce genetic instability leading to high cancer maximum multiplicity in this analysis.

Cluster 1 is relatively heterogeneous, including adenomas, at least one tumor of unknown malignant potential (gastrointestinal stromal tumors), and carcinomas. The mean cancer maximum multiplicity for cluster 1 is considerably lower than cluster 3 (15 compared with 45), and this cluster may not have biological meaning other than a moderate degree of genetic instability. It is interesting to note that the majority of the tumors are from the gastrointestinal tract (esophageal, intestinal of any type, stomach, pancreas, and liver).

## Discussion

Despite its limitations, the COSMIC database is a valuable resource that brings together findings from thousands of studies; our methods demonstrate the extraction of meaningful knowledge representations from this resource. With the onslaught of whole genome analysis data quickly approaching, the need for methods of meaningful knowledge representation grows ever more urgent. We provide a robust method for organizing genome data, derived from decades of cancer research on somatic mutations, into a knowledge representation that is useful for the bench researcher and clinician. Our research demonstrates an organizing principle based on the multiplicity of somatic mutations that could potentially aid researchers and physicians in the discovery of broad-based causative gene mutations versus sporadic mutations within specific tumor types.

The methodology identifies clusters of genes known to drive proliferating cells toward carcinoma. Many of the genes in the central axis (i.e., those with high gene maximum multiplicity values) have a direct relationship to cell cycle regulation. This is consistent with the hypothesis that genes appearing along the central axis are a set of driver genes, given that imbalance between cell promotion and cell death results in tumor progression [7]. By association, other genes in clusters with high gene maximum multiplicity values are potentially driver genes. In contrast, mutations in genes with lower gene maximum multiplicity values may be associated with the environment (anatomic location) and the specific cell type in which they occur. Another measure of multiplicity, signature multiplicity [30], is the phenomenon of multiple optimally predictive signatures of phenotype in high throughput data. The concept of multiplicity introduced in this paper focuses on measuring the systemic occurrence of genes across multiple phenotypes to create clusters. A more extensive comparison between the two multiplicity approaches will be possible when sufficient samples of next-generation sequence data are available on multiple cancer types.

Our research shows that informatics can be used to search data collected from previous studies to discover novel relationships and patterns in the data. With the use of high-throughput technologies that measure the characteristics of well-annotated samples specifically designed to characterize the cancers with multiplicity measures, there is the potential for further confirmatory and new discoveries.

Even given the limitations of the COSMIC data set, there is support for the hypothesis that multiplicity measures can be used to distinguish early from late cancers. The adenomas tend have lower cancer maximum multiplicity than their corresponding carcinomas, consistent with multi-hit model of cancer progression, and particularly applicable for tumors of epithelial origin, where more advanced cancers have more mutations in their DNA. The maximum multiplicity values are reversed for genes, with early causal genes having higher multiplicity values, indicating that early causal genes are systemic across cancers. Gene cluster 4, which has the highest multiplicity value and containing 23 of the 24 genes associated with precancerous tumors gives evidence that the clustering technique separates early initiating genes. Work has been done on mutually exclusive and co-occurring gene pattern in pathways from COSMIC [31], and a next step would be to integrate findings of gene co-occurrence and exclusivity with multiplicity clustering. The bias in the COSMIC data set makes comparing multiplicity clusters and co-occurring genes difficult, but it will be possible with whole genome analysis to assess co-occurrence and multiplicity together. Although the order of gene mutations is stochastic, multiplicity can be used to separate the general progression of these mutations, given sufficient granularity of tumor tissue and genomic measurements.

These multiplicity measures offer a cost-effective way to discover new knowledge using data from previous studies. Additional benefits of the measures will be achieved when sufficient detailed genomic information is available to classify tumors of patients recently diagnosed with a particular cancer. Potentially, distributions of multiplicity measures could be used to characterize severity and type of tumor for individual patients.

### Clinical relevance

In clinical settings, tumors are largely classified according to anatomic location, histological type, subtype, and stage [32]. Histological features, which until now have dominated the subtyping of tumors, do not provide a complete reflection of the molecular alterations that can drive tumor growth and aggressiveness. For example, colonic tumors that penetrate the muscularis, with or without lymph node involvement, have overlapping outcomes. Thus, while microscopic inspection of colonic tumors may be adequate for differentiating benign polyps from malignant growths, it may be inadequate for predicting the aggressive behavior of malignant tumors.

Because the usefulness of localization and appearance for classifying cancers and determining optimal therapies is limited [33], alternative approaches are warranted. Somatic mutations play an important role in cancer initiation and progression, as well as in prognosis [9]. However, biomedical research about somatic mutations has generally been limited to a few genes in a few cancer types. Our informatics methods can augment research into the molecular basis of disease, using data from decades of studies to identify similarities in somatic mutation patterns that characterize similar cancer types. In cases where histological examination produces uncertain diagnoses, individual tumors could conceivably be examined for mutation patterns and compared to a space of tumors constructed from the multiplicity of their somatic mutation patterns. Similar to the situation with overexpression of a molecular target, somatic mutations that have been profiled extensively for some cancer types may provide useful guidance for research into other poorly understood cancer types that have similar somatic mutation patterns.

A tool that identifies patterns in somatic mutations shared across multiple cancer types can be useful to identify a broad selection of cancers that might benefit from molecular diagnostics of one or more somatic mutations coupled with targeted therapy. For example, breast cancers are routinely tested using immunohistochemistry to detect overexpression of the Her-2/neu oncogene; cancers with this overexpression are treated with the targeted therapy trastuzumab. However, tumors with a PI3 kinase (*PIK3CA*) mutation that results in constitutive kinase activity will be resistant to trastuzumab [34]. A number of other inhibitors have been developed that target PI3K activity; some are now being tested in early clinical trials in non-small-cell lung, breast, ovarian, endometrial, brain, and multiple leukemia and lymphoma cancers [35].

Multiplicity analysis of somatic mutations could identify those with the highest multiplicity as those most likely to be effective molecular targets across multiple tumor types. In addition, one can study the impact of associated mutations on the efficacy of novel targeted therapies across multiple tumor types that share mutations.

With the arrival of The Cancer Genome Atlas (TCGA) [36], more detailed information on alterations of the cancer genome is becoming available. The analysis described here can be generalized to other alterations by substituting the multiplicity of somatic mutations with that of copy number, methylation, or microarray data. The data will need to cover many different types of cancer in order to assess the multiplicity of the alterations across cancers.

With a principled organization of whole tumor genomes, both prognostic and predictive testing based on genomic data could become common practice. Identification of somatic mutations, either alone or in combination, could be useful to physicians as a supplement to the histological and immunohistochemical findings currently used to establish diagnosis and prognosis. A multiplicity-based clustering methodology can be a tool to organize the genomic data and to identify somatic mutations occurring in similar sets of cancer.

### Limitation

As the creators of COSMIC have suggested [13], this data set is likely to contain an ethnic bias in which a disproportionate number of samples originated from the United States and Europe. COSMIC also has a prior knowledge bias due to the collection of mutation data focusing on many core cancer genes. The clustering technique generates stable clusters from the three-dimensional multiplicity space and increases in stability as sample across subtypes increases. The number of samples available for each subtype limits the granularity of the disease classification. Therefore, somatic mutations that may be relevant to a particular subtype of a disease will be associated with the larger disease group. This limitation can be addressed in future work as more data become available across subtypes of diseases. These limitations of the data set do not affect the organizing principle of multiplicity and will be resolved as the measure is applied to whole genome data on tumors. Despite these limitations, COSMIC appears to be a valuable resource for developing knowledge representations with potential bench research and clinical relevance.

## Conclusion

Our results demonstrate the power of a method based on the principle of multiplicity to cluster genetic mutations and relate them to the cancer types in which they occur. The concept of multiplicity builds upon Fearon and Vogelstein's multi-hit genetic model of colorectal cancer and generalizes across cancer types. As an organization tool, this approach is helpful in identifying somatic mutations as candidates for targeted therapy that are most likely to impact multiple cancer types, and it identifies associations of mutations that may be informative as to mechanisms of resistance to targeted therapy.

An interesting result of our analysis is that it is more than a solution to the problem of organizing complex genomic data for human interpretation. The results show that genetic mutations known to be causal correlate with high multiplicity. This way of organizing the data lends support to the hypothesis that other genetic mutations with high multiplicity might also have a causal role in the stepwise progression of normal cells to the neoplastic and frankly malignant states. The organization of the data also allows the user to generate hypotheses concerning the role of the tissue type, local anatomic site, or even particular cancer type in promoting genetic mutations that lack high multiplicity.

The concept can be generalized beyond somatic mutations to other modalities such copy number, methylation, and microarray data. The application of the method to these modalities will depend on having sufficient sample across a collection of cancer types. The method's effectiveness at generating meaningful representations from COSMIC forecasts what can be discovered with whole genome analysis of tumor samples. A well-characterized genomic space of genes and cancer types constructed with the principle of multiplicity could be used to compare and cluster patients' tumor samples, potentially resulting in improved diagnostic and prognostic capabilities in clinical practice. It will also be interesting to see how sequence data and expression data correlate. It is not clear that somatic mutations will have a uniform effect on gene expression for the mutated gene, although a somatic mutation in a causative gene may result in a unique gene expression signature. Given the recent investment of the National Cancer Institute in creating TCGA, which contains genomic information from multiple platforms across multiple tumor types, we feel that multiplicity analysis can be utilized to organize the information and engender hypotheses about functional causality from discovered associations.

## Competing interests

The authors declare that they have no competing interests.

## Authors' contributions

All authors contributed to the writing of the manuscript. LJF was the primary author of the manuscript. LJF developed the analysis technique and interpretation. SRP developed software for managing and analyzing the data. MEE interpreted the results from a clinical perspective. All authors read and approved the final manuscript.

## Pre-publication history

The pre-publication history for this paper can be accessed here:

http://www.biomedcentral.com/1755-8794/4/52/prepub

## Supplementary Material

Additional file 1 **Hierarchical clustering for causal germline mutations**. A graphical representation of the hierarchical clustering for these 34 causal germline mutations is made available through Additional file 1. Click here for file

Additional file 2 **Gene maximum multiplicity values for each gene**. As a supplemental resource, a CSV file is included that provides a gene maximum multiplicity value for each gene. Click here for file
