# Supplementary figures and images for: Multiplicity: an organizing principle for cancers and somatic mutations
Source: BMC Med Genomics. 2011 Jun 29;4:52. doi: 10.1186/1755-8794-4-52 (PMC3150236; doi:10.1186/1755-8794-4-52)

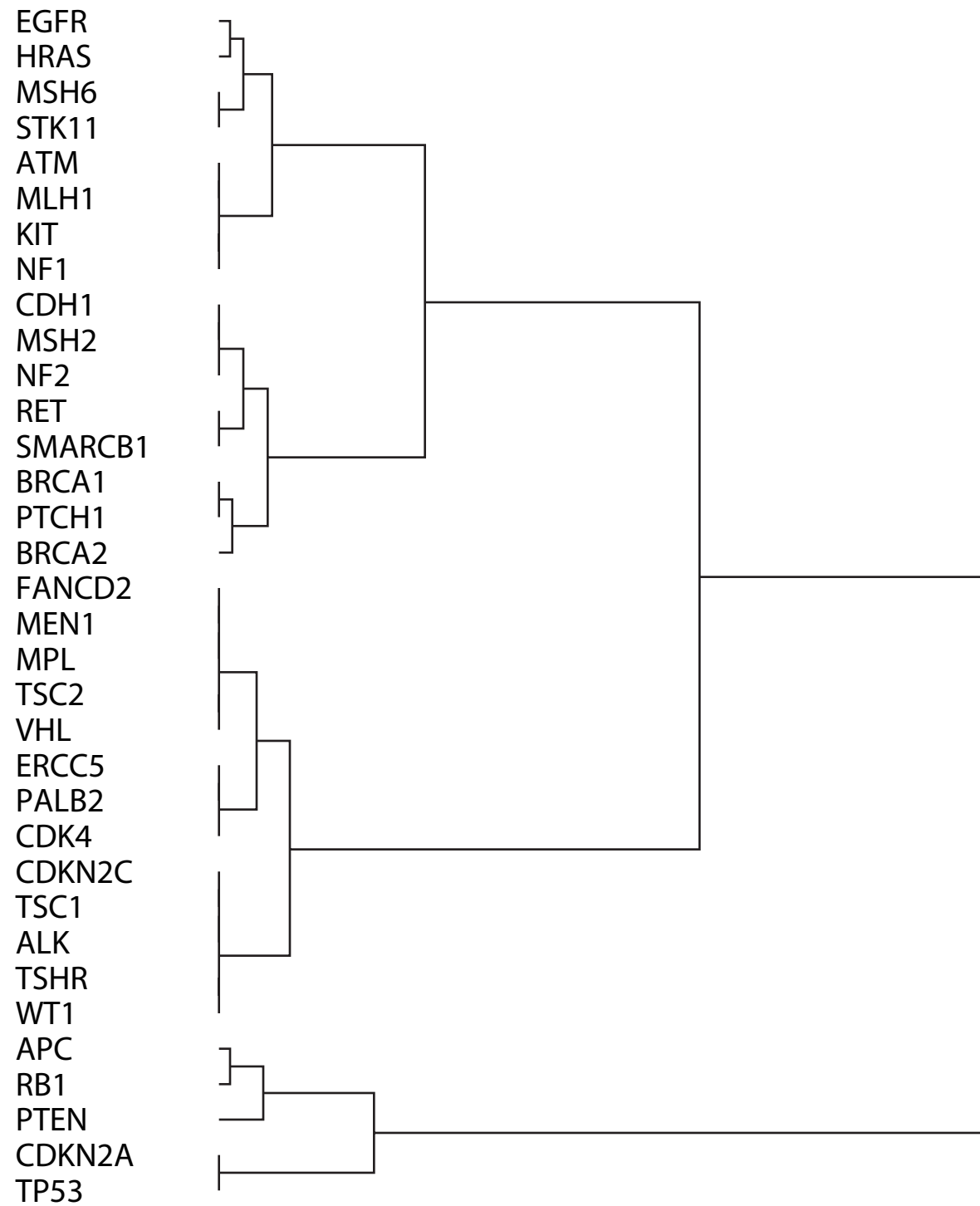

Supplement: Additional file 1 — Hierarchical clustering for causal germline mutations. A graphical representation of the hierarchical clustering for these 34 causal germline mutations is made available through Additional file 1. [file 1755-8794-4-52-S1.PDF]
